# Impact of Social Media on Aesthetic Dentistry: General Practitioners’ Perspectives

**DOI:** 10.3390/healthcare10102055

**Published:** 2022-10-17

**Authors:** Maria Shakoor Abbasi, Abhishek Lal, Gotam Das, Fatima Salman, Aisha Akram, Abdul Razzaq Ahmed, Afsheen Maqsood, Naseer Ahmed

**Affiliations:** 1 Department of Prosthodontics, Altamash Institute of Dental Medicine, Karachi 75500, Pakistan; 2 Department of Prosthodontics, College of Dentistry, King Khalid University, Abha 62529, Saudi Arabia; 3 Department of Oral Pathology, Bahria University Dental College, Karachi 74400, Pakistan; 4 Prosthodontics Unit, School of Dental Sciences, Health Campus, Universiti Sains Malaysia, Kota Bharu 16150, Malaysia

**Keywords:** social media, aesthetic dentistry, dentists, WhatsApp

## Abstract

The objective of this study was to investigate general dentists’ observations of various aesthetic dental procedures among social media users. A cross-sectional study was conducted at the Department of Prosthodontics, Altamash Institute of Dental Medicine, over a duration of six months. Data were collected using a well-structured questionnaire comprising 21 predefined responses. The questionnaire was uploaded and disseminated through Google Surveys by forwarding web page links through emails and WhatsApp. The data collected were analysed through Statistical Package for Social Sciences (SPSS-Version 25). The majority (90.7%) of the dental practitioners surveyed believed that there is an increase in the demand for aesthetic dental procedures and social media is a major contributor to it. Moreover, most of the 377 (81.8%) participants agreed that social media is a beneficial platform enabling them to educate, advertise, and communicate with patients. Concerning popular aesthetic procedures, teeth whitening (54.7%), Hollywood smile (17.1%), dental veneers (11.9%), and Invisalign (10.4%) were the most commonly demanded aesthetic procedures. The patients showed desire for these procedures because they were trending on social media platforms. Almost half of the general dental practitioners used social media to post their content related to aesthetic dentistry and to promote their work. They preferred using before and after pictures (48.6%) for advertising their content related to aesthetic dentistry. Within the limitations of the study, it is concluded that the demand for aesthetic dentistry is rapidly growing, and social media is the main driving force behind this revolution as the general population has direct access to the profiles of celebrities and influencers, who all appear to have ‘the perfect smile’. This shift in people’s self-image has created a surge in patients seeking cosmetic treatments.

## 1. Introduction

Dentistry is a science-based, highly structured healthcare profession that serves progressively more demanding patients [1]. The dental profession was once looked upon as a health sector that only operated to provide treatment of pain, jaws, gums or any other oral cavity diseases. The reason to seek dental care was solely for conventional treatments and patients consulted dentists only when there was an obvious need. Now, with the world changing, it cannot be denied that science and technology has advanced, and this has aided the healthcare system substantially; the medical field has come a long way and dentistry is no exception. Procedures and equipment have evolved extensively [2]. Today, dentistry is not only limited to procedures to treat the ill-fated tooth: elective aesthetic procedures are also being carried out [3].

Aesthetics and physical appearance are imperative attributes that cannot stay unnoticed and contribute to an individual’s personality. A beautiful smile with pearl white teeth framed by perfectly shaped lips makes one appear younger and socially more appealing. Moreover, features such as a defined jaw line, flawless skin, plumped lips, Hollywood smile, contoured nose, and other physically attractive aspects contribute to a person’s confidence level and boost one’s self-esteem [4]. The inclination towards aesthetic dentistry is rapidly growing most certainly because of increased social media usage in the present world, hence making it mandatory for dentists to offer procedures including veneer placements to mimic the Hollywood smile, orthodontic treatment to straighten teeth, and orthognathic surgery when there are extreme dentofacial anomalies that cannot be treated by orthodontic treatment, including corrective bone operations involving mobility of the jawbones. Facial proportions, lip balance, chin−nose balance, nasolabial angle and cheek implants are achieved by orthognathic surgery as well. Moreover, bleaching procedures to whiten teeth, reduction of irregular gum appearance in a smile, Botox to give a fuller look to lips, plasma-rich protein treatments, and many other procedures related to aesthetics are offered in order to satisfy patients: further, the pre-visualisation of the result makes the aesthetic operation more attractive [3,4,5,6,7].

It was observed over time that in some patients, the main aim of any procedure or even orthognathic surgery was to improve their physical attractiveness; extreme abnormalities or deformity made them feel aesthetically vulnerable in the age of social media and they sought social acceptance [5,6,7,8,9]. In this era, social media has the power to modify people’s way of thinking. It does not only showcase an image of beauty but has several passive effects too. Millions of people rely on social media platforms, such as Facebook, Snapchat, YouTube, Twitter, and Instagram to stay updated with the current news [10]. Hegarty et al. conducted a study in which they evaluated the top 60 videos related to orthognathic surgery and found that they had a combined total of 6,986,141 views. Videos predominantly involved patients describing their personal experience (41.67%) with the majority positively biased (61.67%). Moreover, the informative content made up a percentage as small as 9.17%; therefore, it was suggested not to rely on these videos for information and guidance [11]. In contrast, investigations into the impact of media on society have shown that it positively affects a wide variety of other areas, including mental health [12], child development [13], attitudes toward eating habits [14,15], sexual attitudes and behaviors [16,17], violence among children [18], and suicidal tendencies [19]. It is therefore not unreasonable to assume that the media may have a similar impact on aesthetic dentistry. Indeed, in an online survey conducted by American Academy of Cosmetic Dentistry (AACD), dentists cited media coverage as the main reason for the increase in demand (by an average of 12.5% over the previous five years, with some dentists reporting an increase of almost 40% for aesthetic dental procedures) [20].

However, media exposure may not always portray dentistry in a positive light: Rachel Henzell conducted a study to qualitatively analyse the orthodontic-related posts on Twitter and found mixed opinions. For example, some of the users expressed great excitement and relief about getting their braces removed soon whereas some used the hashtags “#Ow” and “#KillMe” to illustrate the extreme nature of their feelings [21]. Additionally, Arab et al. [22] conducted a quantitative observational cross-sectional study using a self-constructed questionnaire to evaluate the influence of viewing social media advertisements related to cosmetic surgery and found that 48.5% reported being influenced by social media to consider undergoing cosmetic procedures. They had a negative self-view when viewing social media for more than 5 h per day. Thus, social media’s influence on aesthetic dentistry can be both positive and negative. To date, little is known about the demand for aesthetic dentistry in Pakistan. The present study is the first to consider the impact of social media on dental practice, promotion and professionalism among general dental practitioners and specialists in Pakistan: This matter may be of use to emerging dentists or new technologies.

This study aimed to investigate whether social media have affected the perception of, and demand for, aesthetic dentistry in Pakistan.

## 2. Materials and Methods

### 2.1. Study Setting and Ethical Approval

This cross-sectional study was conducted at the Department of Prosthodontics, Altamash Institute of Dental Medicine, over a duration of six months, October 2021–March 2022. Participants’ consent was sought and ethical approval was received from the ethical review committee (AIDM/RDRC/06/2021/05).

### 2.2. Study Protocol

The sample size was calculated using the WHO sample size calculator, considering the mean value of 59% [5] for social media beneficence. The confidence interval was 95% and the margin of error 5%, and the power of study 80. The estimated sample size for this study was 461 participants. General practitioners of either gender, currently practicing and with at least 1 year of clinical experience were included in the study. House officers or non-practicing dentists were excluded.

### 2.3. Study Questionnaire

Data were collected using a well-structured questionnaire. A pilot study was conducted on 40 participants to validate the questionnaire. The internal consistency of items tested with intra-class correlation showed a strong value of 0.839.

A well-structured questionnaire was used for data collection. A consent statement for voluntary participation and declaration of anonymity and confidentially were included in the questionnaire for all subjects to understand prior to their agreement. The questionnaire comprised 21 predefined responses including demographic, social media usage, and aesthetic dentistry-related questions. The questions that were included in the questionnaire were: which is the most used social media platform, how many hours do you spend on social media, how much of your activity is centered around aesthetic dentistry, do you post about your aesthetic procedures and if not, why not. Moreover, it asked whether the increased use of social media also increased the demand for certain procedures, whether social media increased knowledge about aesthetic procedures, and if social media had increased knowledge and awareness of aesthetic dentistry among their patients.

**Figure 1:**
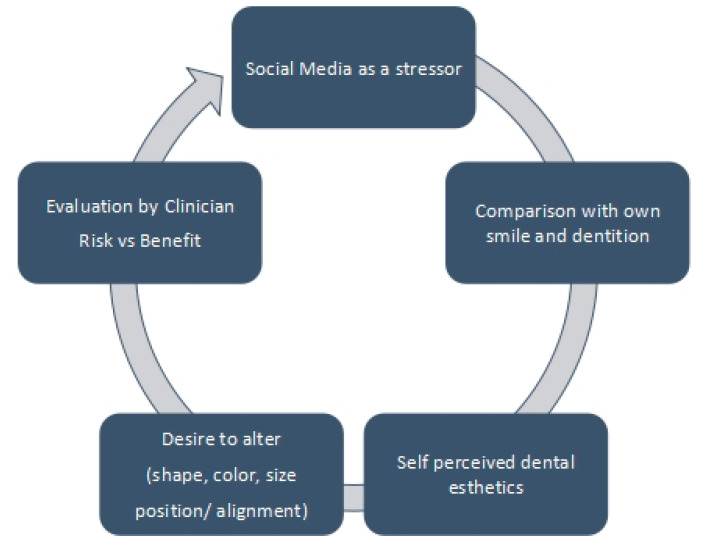
The impact of social media on dental aesthetic alteration.

The participants were also asked if they believed that social media was beneficial in educating and communicating with their patients; furthermore, dentists had to vote for the most popular aesthetic procedure that was in demand. Another question was whether a patient had ever shown interest in a particular aesthetic procedure because of any social media trend, what was the most catchy mode of advertisement which drew the patient’s attention, and whether social media was a beneficial platform for educational and advertisement purposes. Furthermore, if the participants did not agree with social media being an effective tool to advertise and educate, they were then asked what was the reason for this and had something changed their mindset. The dental practitioners were also asked if they practiced editing their photos before posting them on social media. The last question covered in the questionnaire was whether the information provided on social networking sites about aesthetic dentistry was enough to gain knowledge and satisfy patients or not. The summary of the questionnaire is depicted in Figure 1.

The questionnaire was then uploaded at www.surveys.google.com (accessed on 13 August 2021).The online survey link was circulated through social media (WhatsApp, Facebook, Twitter) and emailed all over Pakistan.

### 2.4. Statistical Analysis

The data collected were analysed through Statistical Package for Social Sciences (SPSS-Version 25). The descriptive statistics analysis was carried out to calculate the mean and standard deviation of continuous variables and the percentage values of categorical variables. Linear regression analysis was carried out to detect an association between the demographic details of participants and social media impact responses. A *p*-value of ≤0.05 was taken as significant.

## 3. Results

In this cross-sectional study, a total number of 498 dental practitioners participated in the study from all over Pakistan and submitted the questionnaire. Thirty-seven forms were rejected due to partially filled questionnaires or incorrect information. Finally, a total of 461 general practitioners was included in the final study.

The majority of the participants were in the age brackets of 26–30 years (65.9%), and 31–35 years (27.5%). Regarding gender distribution, 199 (43.2%) males and 258 (56.0%) females participated in this study. Regarding the residential province, the majority of the participants were from Sindh (64.9%), and Khyber Pakhtunkhwa (21.0%). With respect to the marital status of the participants, 279 (60.5%) were unmarried and 182 (39.5%) were married. Regarding the years of practice, most of the participants had practiced dentistry for 1–5 years (49.2%), and 6–10 years (26.0%), as presented in Table 1.

**Figure 2:**
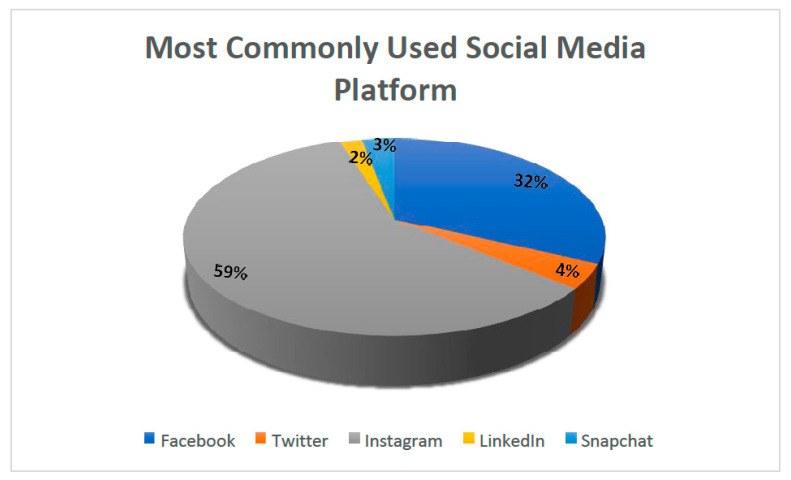
Social media platforms most commonly used by the participants.

Regarding the most frequently used social media platform, most of the general practitioners used Instagram^©^ (59.2%), followed by Facebook^©^ (31.7%), with a small number of participants using Snapchat^©^, Twitter^©^, and LinkedIn^©^, as presented in Figure 2. Regarding the number of hours spent on social media, most of the respondents used social media for 2–3 h (30.2%), 3–5 h (27.5%), and 1–2 h (22.8%). On the search activity of the term aesthetic dentistry in search engines, the term aesthetic dentistry most commonly contributed 30–40% (40.3%), 10–20% (24.9%), and 50–60% (17.8%) to the total search of the participants.

With reference to posting content related to aesthetic dentistry, 56.8% of the general practitioners answered “yes”, and 42.5% answered “no”. Furthermore, the reason for not posting content related to aesthetic dentistry was the majority believed that they “don’t have time” (37.3%), followed by “ethical concerns” (21.0%) about posting such content, and ”avoiding negative feedback” (8.0%). However, about 95 (20.6%) of the general practitioners did post content related to aesthetic dentistry.

With the recent increase in patients’ knowledge and awareness regarding aesthetic dental procedures, most of the 418 (90.7%) dentists believed that there was an increase in the demand for aesthetic dental procedures. Moreover, many general practitioners (95.4%) noted an increase in knowledge of aesthetic dental procedures among the patients. Regarding the impact of social media on the increase in knowledge and awareness regarding aesthetic dentistry, the majority of the 313 (67.9%) general practitioners believed social media had a major contribution to it. Moreover, most of 377 (81.8%) of the participants agreed that social media was a beneficial platform allowing them to educate, advertise, and communicate with patients.

**Figure 3:**
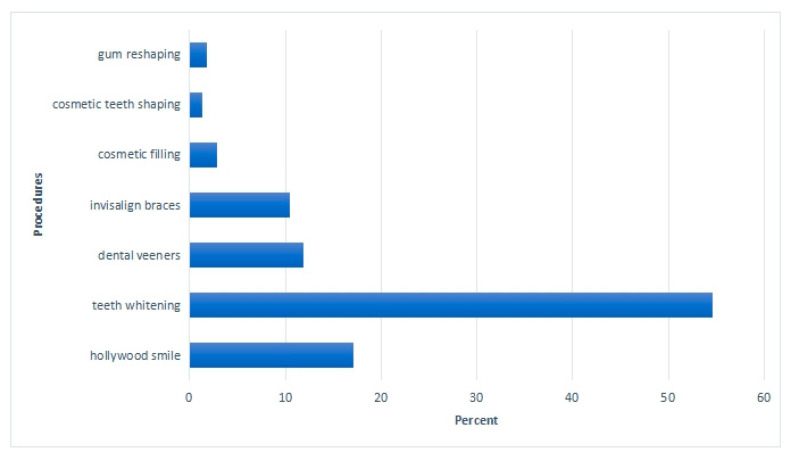
Aesthetic dental procedures most commonly demanded by patients.

About the popular aesthetic procedures, teeth whitening (54.7%), Hollywood (17.1%), dental veneers (11.9%), and Invisalign (10.4%) were the most commonly demanded aesthetic procedures by the patients, as presented in Figure 3. Many of the dentists (59.4%) and general practitioners believed that the patients that visited their dental practices wanted to have aesthetic dental procedures performed as they were trending on social media.

Regarding the mode of advertisement to attract patients towards aesthetic dental treatments, the general practitioners preferred using before and after pictures (48.6%), family and friends’ referrals (21.9%), and online/social media advertising (18.7%). Regarding whether advertising was beneficial, the majority of the participants answered: “yes” (76.1%), and “maybe” (19.5%). However, some participants believed advertising not to be beneficial because of the following reasons: limited information (12.8%), biased environment (11.1%), manipulative content (8.2%), and fake reviews (5.4%). In reference to editing the pictures captured by the dentists, the majority of 318 (69.0%) respondents answered “no” to using photo editing applications to edit the pictures, with 101 (21.9%) agreeing to having edited the photos. About the information available on social media regarding aesthetic dental procedures, 160 (34.7%) responded “yes” that sufficient information was available, whereas 175 (38.0%) answered “no”, and 126 (27.3%) answered “maybe”.

The demographic characteristics such as age, gender, province of residence, years of practice, and marital status of the participants were compared with the responses of the participants using multiple linear regression analysis. It was found that age (*p* = 0.001), province (*p* = 0.002 and years of practice *(p* = 0.001) had a significant relationship with the responses of the participants, as presented in Table 2.

## 4. Discussion

This study is focused on the evaluation of and demand for aesthetic procedures driven by social media in a dental practice and how dentists are making the most of social media platforms to market their skills and to promote the aesthetic trends. As they are easily accessible, constantly updated, and globally available to anyone with a network connection, social media sites such as Facebook, Instagram, Twitter, Snapchat, LinkedIn, and many others have tremendous power to influence the mindsets of people [22]. The American Society for Aesthetic Plastic Surgery reported that after the advent of social media in 1997, cosmetic procedures in the United States had increased by 446%. In 2006 alone, there were nearly 11.6 million cosmetic procedures performed in the United States [23]. According to Sarwer et al., three potential explanations exist for this marked increase in the popularity of aesthetic procedures: (1) recommendations by, and availability of, medical practitioners; (2) mass media and the entertainment industry; and (3) factors personally related to patients [24]. Literature shows that successful practitioners of medicine, dentistry, cosmetic or otherwise, rely on reputation on social media to establish and expand their practice and patient base. They have an undeniable impact on the supply and demand for aesthetic procedures offered by dental practitioners [25,26,27].

Aesthetic dentistry, in particular, is an art in which subjective perception of the outcome of cosmetic procedures drives patient satisfaction. Understanding market trends has important implications for practitioners’ training and choice of dental aesthetic trends inventory [26]. In the present study, the majority of participants reported that social media had an effect on their satisfaction with the appearance of their teeth. According to our research survey, the most commonly used social media platform was Instagram. However, in contrast, Bahabri et al. [28] conducted an online survey and found that Facebook was the platform most commonly used among dentists to promote their services. Similar findings were observed by Parmar et al., who found Facebook was the most popular platform to use for both patients (98%), and dentists (77%). For patients, Twitter, Instagram, and LinkedIn were popular choices in their personal lives [29]. This information is important as it could assist dental practitioners in determining which platform has the greatest impact in their region and is, thus, the most suitable for the publication and marketing of their skills. In the present study, 56.8% of dentists agreed that they used social media apps to post aesthetic procedures. This percentage is in accordance with the study conducted by Salim et al., where 57% of dentists reported posting affirmative content related to aesthetic dentistry. Further, 37% of dentists reported that they did not post content on social media because of the lack of time while a few said that it was due to ethical concerns, which is in accordance with the study conducted in the Kingdom of Saudi Arabia (KSA) where dentists stated similar reasons for not posting content related to dentistry on their social media platform [30]. In addition, the majority of dentists (90%) observed a rise in demand for aesthetic procedures in their dental practice which is in accordance with a study conducted in the Gulf region where (53%) of the patients were following a dentist or a dental clinic on social media, and they significantly agreed that dentists should communicate with people through social media rather than conventional media. This is in agreement with other Western studies that found patients appreciated the presence of healthcare providers in social networks [31,32]: the patients thought it was tough to choose a dental practice, which was made easier by digital social media apps.

Moreover, there are multiple ways via which dentists can advertise their clinical skills to reach patients easily. In the present study, 50% of the general dentists reported that before and after pictures of the procedures drew the attention of patients toward their practices. This might be because Instagram was the widely used social media platform in our survey. Moreover, picture representation aids visualisation of the pre-treatment and post-treatment state of a procedure. The majority of the dental practitioners agreed they did not use any type of editing app before posting pictures of cases on social media while some confirmed the use of editing apps. This is contrary to the study conducted in KSA, where most of the dentists reported editing pictures before uploading them on social media [26]. This might be because of the cultural difference in both countries as marketing dental procedures and posting cases is yet not very popular in our region compared to KSA. With the rise of social media, there has been a dramatic increase in demand for cosmetic dental treatments. Through social media, there is direct access to profiles of celebrities and influencers, who all appear to have ‘the perfect smile’. These shifts in people’s self-image have created a surge in patients seeking cosmetic treatments [7], and 95% of the dentists agreed that social media has contributed to their patients’ knowledge and awareness of aesthetic dentistry, which is in accordance with another study where social media was the means of sharing and receiving information for most patients (54.3%) [26]. In the present study, 82% of the participants were in agreement that social media was a beneficial platform allowing them to educate, advertise, and communicate with patients, which is in accordance with a study conducted in the Gulf where 46.3% of dental professionals, and 65% of patients mentioned that social media platforms could be mainly used for both marketing and community services. Furthermore, 37.6% of dental professionals clarified that sometimes they had the ability to answer queries and offer consultation to their followers [26]. While 81% of participants considered it beneficial, the other 12.6% said that there was a provision of limited information and knowledge on social media apps. This might be due to the fact that social media might hold incorrect or incomplete information which might be misleading.

The popularity of aesthetic dental procedures is due to the increased usage of social media in daily life. It influences people to follow the current beauty or aesthetic trends [33,34,35]. Sixty-one percent of the dentists confirmed that patients inquired about aesthetic procedures after being influenced by social media trends; 54.7% of dental practitioners said that the most popular aesthetic procedure in demand was teeth whitening. In a study by Ajwa et al. [35], more than half of the patients (56.2%) wanted cosmetic treatment as they were not happy with the color of their teeth. Tin-Oo et al. [36] found that the general public was dissatisfied with relatively mildly discolored teeth, indicating their concern about the color of their teeth, and tooth whitening was the treatment most desired by patients (48.1%). The second most popular procedure in demand on social media was the Hollywood smile, voted by 17.5% of dentists in our survey. A “Hollywood smile” can be described as an appropriate positioning of teeth, labial and gingival tissues within the dynamic display zone. This aesthetically perfect smile is achieved by multiple procedures, including smile analysis, designing a trial smile, and correcting by special means (a combination of all or some of the following: tooth whitening, crowns, veneers, and orthodontics). More than half of the dentists (61%) agreed that after being influenced by the media, patients do ask about specific aesthetic procedures as they strive to be the perfect version of themselves. Moreover, the main reason behind this inspiration is the trends being set on social media. Therefore, there is a critical need to educate the general population and enhance awareness.

The limitations of our study were firstly, the minimized access to every region of the country. Secondly, the responses of the participants may have varied according to the time spent on social media. Lastly, the responses of the participants that did not use social media were not included. In the future, a study with a greater sample size targeting the different regions of Pakistan can be undertaken with the inclusion of other social media applications that are emerging each day and being used by practitioners.

## 5. Conclusions

Within the limitations of the study, it is concluded that the demand for aesthetic dentistry is rapidly growing, and social media constitutes the main driving force behind this revolution as the general population has direct access to the profiles of celebrities and influencers, who all appear to have ‘the perfect smile’. This shift in people’s self-image has created a surge in patients seeking cosmetic treatments. Therefore, there is a critical need for effective and repeated education alongside a widespread public campaign aiming to enhance relevant awareness and contemporary information related to social media applications.

## Figures and Tables

**Table 1:** Distribution of demographic details of the participant (*n* = 461).

Variables		Frequency and Percentage
Age	26–30 years	304 (65.9%)
	31–35 years	127 (27.5%)
	36–40 years	12 (2.6%)
	46–50 years	14 (3.0%)
	51–55 years	4 (0.9%)
Gender	Male	199 (43.2%)
	Female	258 (56.0%)
Marital Status	Married	182 (39.5%)
	Unmarried	279 (60.5%)
State	Sindh	299 (64.9%)
	Punjab	43 (9.3%)
	Balochistan	18 (3.9%)
	Khyber Pakhtunkhwa	97 (21.0%)
	Federally Administered (Islamabad)	4 (0.9%)
Years of Practice	<1 year	76 (16.5%)
	1–5 years	227 (49.2%)
	6–10 years	120 (26.0%)
	11–24 years	25 (5.4%)
	25 or more years	13 (2.8%)

**Table 2:** Association of age, gender, province of residence, years of practice, and marital status with responses of the participants.

Variables	Unstandardized Coefficients		Standardized Coefficients	*t*	*p*-Value
Age	**B**	**Std. Error**	**Beta**		
	0.039	0.029	0.061	1.337	0.001
Gender	0.226	0.030	0.535	7.514	0.182
Province	0.046	0.015	0.142	3.061	0.002
Years of Practice	−0.137	0.027	−0.382	−5.133	0.001
Marital status	0.011	0.029	0.018	0.390	0.697

## Data Availability

The data included in the present study are available upon request from the corresponding author.
